# Utilization of serum D-dimer assays prior to computed tomography pulmonary angiography scans in the diagnosis of pulmonary embolism among emergency department physicians: a retrospective observational study

**DOI:** 10.1186/s12873-021-00401-x

**Published:** 2021-01-19

**Authors:** Leila Salehi, Prashant Phalpher, Hubert Yu, Jeffrey Jaskolka, Marc Ossip, Christopher Meaney, Rahim Valani, Mathew Mercuri

**Affiliations:** 1 Department of Family Medicine, McMaster University, 100 Main Street West, 6th floor, Hamilton, Ontario Canada; 2 Department of Family and Community Medicine, University of Toronto, 500 University Avenue, Toronto, Ontario Canada; 3 Department of Emergency Medicine, William Osler Health System, Suite S.1.184, 2100 Bovaird Avenue East, Brampton, Ontario Canada; 4 Department of Diagnostic Imaging, William Osler Health System, 2100 Bovaird Avenue East, Brampton, Ontario Canada; 5 Division of Emergency Medicine, McMaster University, Hamilton Health Sciences, McMaster Clinic, 2nd floor, 237 Barton Street East, Hamilton, Ontario Canada

**Keywords:** Pulmonary embolism, Physicians practice patterns, Computed tomography, Biomarkers, Quality improvement

## Abstract

**Background:**

A variety of evidence-based algorithms and decision rules using D-Dimer testing have been proposed as instruments to allow physicians to safely rule out a pulmonary embolism (PE) in low-risk patients.

**Objective:**

To describe the prevalence of D-Dimer utilization among emergency department (ED) physicians and its impact on positive yields and utilization rates of Computed Tomography Pulmonary Angiography (CTPA).

**Methods:**

Data was collected on all CTPA studies ordered by ED physicians at three sites during a 2-year period. Using a chi-square test, we compared the diagnostic yield for those patients who had a D-Dimer prior to their CTPA and those who did not. Secondary analysis was done to examine the impact of D-Dimer testing prior to CTPA on individual physician diagnostic yield or utilization rate.

**Results:**

A total of 2811 CTPAs were included in the analysis. Of these, 964 CTPAs (34.3%) were ordered without a D-Dimer, and 343 (18.7%) underwent a CTPA despite a negative D-Dimer. Those CTPAs preceded by a D-Dimer showed no significant difference in positive yields when compared to those ordered without a D-Dimer (9.9% versus 11.3%, *p* = 0.26). At the individual physician level, no statistically significant relationship was found between D-Dimer utilization and CTPA utilization rate or diagnostic yield.

**Conclusion:**

This study provides evidence of suboptimal adherence to guidelines in terms of D-Dimer screening prior to CTPA, and forgoing CTPAs in patients with negative D-Dimers. However, the lack of a positive impact of D-Dimer testing on either CTPA diagnostic yield or utilization rate is indicative of issues relating to the high false-positive rates associated with D-Dimer screening.

## Background

The correct and timely identification of pulmonary embolism (PE) in the emergency department (ED) remains a major diagnostic challenge for emergency physicians, due largely to its non-specific clinical presentation, particularly in patients with multiple respiratory and cardiac comorbidities, and its potentially lethal outcomes if undiagnosed and untreated. Computed Tomography pulmonary angiography (CTPA) is a very sensitive and non-invasive imaging modality, and has been an invaluable tool in the diagnostic work-up and management of patients with suspected PE.

As the availability and ease of access of CTPA in emergency departments increased over the past two decades, so too did its utilization by emergency physicians [1–4]. This higher utilization has, over time and across different centres, been associated with substantially lower diagnostic yields [1, 4–7]. The mounting evidence thus suggests a phenomenon of overutilization of CTPA among physicians in general, and among ED physicians in particular [2, 3, 8–10].

This has given rise to concerns about both the increased resource utilization and health care costs, the potential harms of exposure to radiation and contrast dye to patients, and the potential harms of ‘overdiagnosis’, i.e. diagnosis and subsequent treatment of clinically insignificant disease [3, 11–14].

Multiple evidence-based algorithms and decision rules have been proposed, developed and tested with the goal of optimizing ordering behaviour among physicians and stemming the overutilization of CTPAs. These algorithms function primarily by allowing physicians to safely rule out pulmonary embolism in low-risk patients based on clinical criteria and/or serum assays, namely the D-Dimer assay, thus obviating the need for CT imaging as a diagnostic modality [15–19]. The initial controlled trials examining the impact of such diagnostic algorithms demonstrated the potential for these to decrease CTPA utilization without increasing risk of missing the diagnosis of pulmonary embolism [18, 20, 21]. However, subsequent studies in less controlled real-world settings have shown less promising results, with variability in physician uptake of these decision rules, and overall inter-physician variability in practice patterns as they relate to both utilization and diagnostic yield of CTPA [17, 22–26].

The objective of this study is twofold. First, we aim to describe the use of D-Dimer assays by ED physicians as a screening test prior to ordering a CTPA for patients suspected of having a PE. Second, we aim to describe the impact of D-Dimer utilization on both CTPA diagnostic yields and utilization rates at our institution over a two-year period.

## Methods

### Study site

We conducted a retrospective observational study of two very high-volume community emergency departments and one high-volume urgent care centre (which opened on February 8th, 2017), all within the same health care system (see Table 1 for study site characteristics derived from our institution’s annual Fiscal Year reports).

**Table 1:** Study site characteristics for the 2017–18 Financial Year (April 1st, 2017 to March 31st, 2018)

Study site characteristics	Community hospital (Site A)	Community hospital (Site B)	Urgent Care Centre (Site C)
Hospital type	Community – Large Hospital	Community – Large Hospital	Community – Urgent Care Centre
Total number of acute care beds^a^	406	230	N/A
Annual ED visit	135,134	82,717	61,949
Admission rate	12.6%	14.4%	1.2%
Total number of admissions from the ED	17,068	11,882	772

^*a*^ *Acute care beds exclude mental health, rehabilitation, and alternate level of care (ALC) beds*

### Data collection

We identified all CTPAs ordered by emergency physicians between January 1, 2016 and December 31, 2017. We then collected clinical and demographic data on all patients who underwent CTPA testing ordered by an emergency physician. This data was collected through our institution’s Radiology Information Systems (RIS), our Electronic Medical Records system and the National Ambulatory Care Reporting System (NACRS). The methodology for data collection has been published elsewhere [27]. The collected variables were: age, sex, chief complaint, Canadian Triage and Acuity Scale (CTAS) level, any serum D-Dimer assay values obtained in the 24 h prior to undergoing the CTPA, and the radiologists’ report of the CTPA findings. The D-Dimer assay used at our institution is the HemosIL D-dimer High Sensitivity assay. The CT scanners in use at the time of the study were the Siemens Somatom Sensation 64 (64 slice scanner) and the Siemens Somatom Definition dual source scanner (tube A 64 slice, Tube B 20 slice).

All CTPA reports were reviewed, and labelled as either positive or negative for a pulmonary embolism. The review was limited to the radiologists’ reports, and did not involve review of the original CTPA images. The data abstraction was carried out by a research assistant who underwent training by the PI (LS) in review of the radiologists’ reports and categorization of the CTPA studies into either ‘positive’ or ‘negative’ for PE. All reports that did not clearly report the finding of a positive or negative PE, and all reports wherein the research assistant was unsure of the appropriate categorization, were reviewed by the PI. The research assistant was not blinded to the study objective and methodology.

All findings of thromboembolic disease (including subsegmental and chronic pulmonary embolisms) were labelled as positive for PE. All inconclusive CTPA studies were followed up by a dedicated search of the patient’s electronic medical record to determine if follow-up investigations, assessment by consultants, or future ED visits yielded a diagnosis of PE. For each CTPA, we also determined if 1) a D-Dimer had been ordered prior to the CTPA, and 2) if the D-Dimer was positive (≥ 230 μg/L DDU).

Of note, our institution’s Department of Laboratory Medicine does not currently report D-dimer values adjusted for age or in accordance with any other probability-adjusted approach, nor does the Laboratory endorse any such modification. The use of such probability-adjusted cut-off values is at the discretion of the treating physician. Therefore, D-Dimer positivity was determined based on a single cut-off value of ≥230 μg/L DDU.

### Data analysis

In the primary analysis of the data, prevalence of D-Dimer utilization prior to the ordering of a CTPA, as well as diagnostic yields of CTPAs ordered, were calculated using simple descriptive statistics. The diagnostic yields for the group of patients who had a D-Dimer prior to their CTPA and those who did not were compared using a chi-square test.

We conducted further secondary analyses into each physician’s individual ordering behaviour, with respect to their use of D-Dimer testing prior to the ordering of CTPA. We used the proportion of CTPAs that were ordered after a *positive* D-Dimer, rather than strict formal risk-stratification according a clinical prediction score such as the Geneva score or the Wells score, as a surrogate measure for the degree of adherence by each physician to determining an objective pre-test probability prior to ordering a CTPA. In this calculation, the denominator contains *both* the total number of CTPAs ordered after a *negative* D-Dimer, and the total number of CTPA ordered *without* a prior D-Dimer.

$$ \mathrm{Guideline}-\mathrm{concordant}\ \mathrm{D}-\mathrm{Dimer}\ \mathrm{utilization}=\left[\ \mathrm{CTPA}\ \mathrm{ordered}\ \mathrm{after}\ \mathrm{a}\ \mathrm{positive}\ \mathrm{D}-\mathrm{Dimer}\ \right]/\left[\mathrm{CTPA}\ \mathrm{ordered}\ \mathrm{after}\ \mathrm{a}\  positive\ \mathrm{D}-\mathrm{Dimer}+\mathrm{CTPA}\ \mathrm{ordered}\ \mathrm{after}\ \mathrm{a}\  negative\ \mathrm{D}-\mathrm{Dimer}+\mathrm{CTPA}\ \mathrm{ordered}\  without\ \mathrm{a}\ \mathrm{prior}\ \mathrm{D}-\mathrm{Dimer}\right] $$

The rationale behind this decision is fourfold. First, our data on patients’ Wells score classification was unreliable and of poor quality. Though our sites do mandate risk stratification of patients according to the Wells criteria prior to ordering a CTPA, it is possible to over-ride or circumvent these as dictated by an individual physician’s clinical judgement. As well, given that there is no way to independently corroborate the different criteria of the Wells score reported by each physician, it is possible for physicians to simply over-estimate and over-report the patient’s Wells score. Furthermore, the determination of a patient’s Wells score is itself based on subjective criteria such as ‘Clinical signs and symptoms of DVT’ and ‘PE is most likely diagnosis’, rendering objective interrater reliability problematic [28]. Finally, the question of whether or not the Wells score adds value above and beyond simple clinician gestalt to the determination of a patient’s pre-test probability for PE remains an issue of debate [29]. Therefore, we elected to only examine the variable of guideline-concordant D-Dimer utilization (i.e. ordering of a CTPA only after a *positive* D-Dimer), as this is more reflective of D-Dimer utilization in real-world practice settings.

Additional measures for each individual physician included 1) the physician’s diagnostic yield, defined as the proportion of CTPAs ordered by that physician that were positive for a pulmonary embolism, and 2) the physician’s CTPA utilization rate, defined as the number of CTPA ordered per 1000 ED visits. We conducted unadjusted linear regression analysis in order to determine if, within our population of ED physicians, greater adherence to the practice of guideline-concordant D-Dimer utilization prior to CTPA was associated with either of the desired outcomes of greater diagnostic yields or lower utilization rates.

All analyses were conducted using SAS statistical software program version 9.4 for Windows, SAS Institute Inc. and Excel Office 2016.

Ethics approval was obtained through the William Osler Health System Research Ethics Office.

## Results

A total of 2824 CTPAs were ordered by a total of 91 ED physicians during the 2-year study period. Of these, demographic and clinical data were available for 2811 studies ordered by a total of 85 physicians. Overall 292 CTPAs out of 2811 (10.4%) were positive for a PE.

Table 2 shows the patient population characteristics of those patients who had a CTPA and for whom clinical and demographic data was available.

**Table 2:** Clinical and demographic characteristics of those patients who underwent a CTPA during the study period

	Overall (***N*** = 2811)	PE positive (***N*** = 292)	PE negative (***N*** = 2519)
**Age (mean, SD)**	59.7 (17.3)	60.2 (15.5)	59.7 (17.5)
**Gender (N, %)**			
M	1112 (39.6%)	152 (52.1%)	960 (38.1%)
F	1699 (60.4%)	140 (47.9%)	1559 (61.9%)
**CTAS score**^**a**^			
1	45 (1.6%)	7 (2.4%)	38 (1.5%)
2	1855 (66.1%)	205 (70.5%)	1650 (65.6%)
3	888 (31.7%)	78 (26.8%)	810 (32.2%)
4,5	18 (0.6%)	1 (0.3%)	17 (0.7%)
Not available	5	1	4
**Chief Complaint**			
chest pain	968	83	886
shortness of breath	786	81	705
syncope/presyncope	105	13	92
other	947	114	832
Not available	5	1	4

^a^CTAS scores are the Canadian Triage and Acuity Scale scores, wherein CTAS 1 is highest acuity and CTAS 5 is lowest acuity

**Fig. 1:**
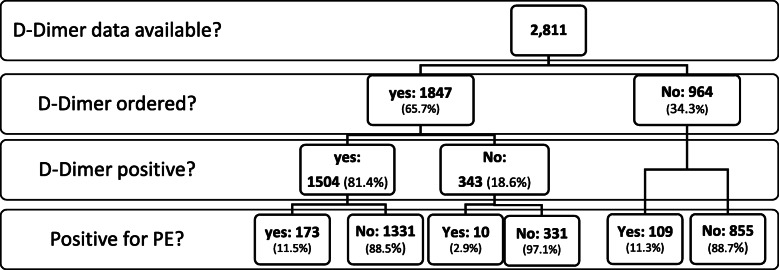
Flow chart of D-Dimer tests ordered prior to CTPA

Figure 1 shows the D-Dimer results and CTPA positive yield results for those patients for whom both CTPA data and D-Dimer data was available.

Ten of the 373 CTPAs ordered after a negative D-Dimer were positive for a pulmonary embolism. Two of these were repeat CTPAs done on the same patient one week apart. Though not part of the original study methodology, we carried out a detailed chart review of these nine patients. All patients were assessed by a hematologist, and in seven out of nine cases, treatment (or treatment change if the patient was already on anticoagulation) was recommended. Of these seven cases, all CTPAs demonstrated lobar or segmental PEs. Four of the seven were recurrent PEs. In the other two cases, the patient was already on anticoagulation and was advised to continue, i.e. the new findings were judged by the hematologist to either be chronic pulmonary emboli not requiring treatment change or not clinically significant.

A total of 964 CTPA studies (34.3% of the total number of CTPAs) were performed without a prior D-Dimer. When compared as a group, those CTPAs preceded by a D-Dimer showed no significant difference in positive yields when compared to those CTPAs ordered without a prior D-Dimer (9.9% versus 11.3%, *p* = 0.26).

Secondary analysis to determine the impact of D-Dimer ordering on both positivity yields and ordering rates at the individual physician level was limited to 85 physicians who ordered a total of 2807 CTPAs. The number of CTPAs ordered per physician during the study period ranged from 1 CTPA to 231 CTPA (median: 18, IQR: 35). Distribution of CTPA utilization rate among the ED physicians ranged from 0.9 CTPA per 1000 ED visits to 22.2 CTPA per 1000 ED visits (median: 4.8, IQR: 4.5). Distribution of positive diagnostic yield ranged from 0 to 50% (median: 9%, IQR: 12.2%). The percentage of CTPAs ordered by each physician after a positive D-Dimer (guideline-concordant D-Dimer utilization) ranged from 0 to 100% (median: 54%, IQR: 29.2%).

**Fig. 2:**
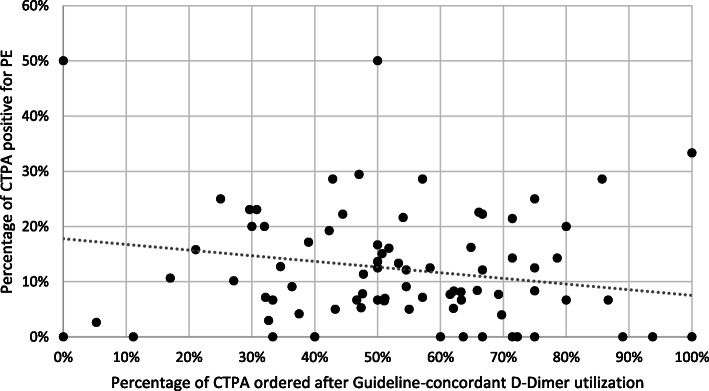
The relationship between the percentage of CTPA ordered after a positive D-Dimer and each physician’s diagnostic yield

Figure 2 illustrates the relationship between guideline-concordant D-Dimer utilization and diagnostic yield for the physicians in the ED group. Each dot represents one physician. On the X-axis is the percentage of CTPAs ordered by the physician after a positive D-Dimer (i.e. the percentage of guideline-concordant CTPAs ordered), and on the Y-axis is each physician’s diagnostic yield (i.e. the percentage of CTPAs ordered by that physician that were positive for a PE).

**Fig. 3:**
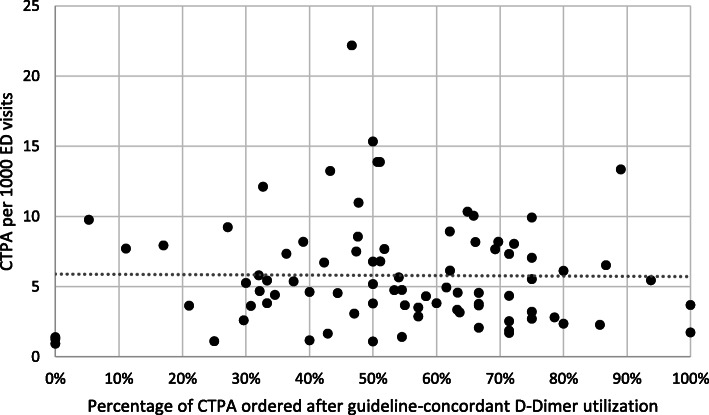
The relationship between the percentage of CTPAs ordered after a positive D-Dimer and each physician’s CTPA utilization rate

Figure 3 illustrates the relationship between guideline-concordant D-Dimer utilization and ordering rate (i.e. the number of CTPAs ordered by that physician per 1000 ED visits) for the physicians in the ED group.

Unadjusted regression analysis revealed no significant association between guideline-concordant D-Dimer utilization and positive diagnostic yield (*p* = 0.0662), nor between D-Dimer ordering and CTPA utilization rate (*p* = 0.992) within the group of physicians.

## Discussion

The findings of this study present a complicated picture of the impact of D-Dimer utilization on CTPA ordering patterns. Of those patients who underwent D-Dimer testing prior to CTPA, 18.7% underwent a CTPA despite a negative D-Dimer. This high prevalence of CTPA ordered on otherwise low-risk patients is not unique to our site, and has in fact been reported in similar rates in other studies [30, 31]. It should be noted that our results did show a slightly higher proportion of clinically significant pulmonary emboli (2.9%) among D-Dimer negative patients than has been found in previous similar studies [8, 30–35]. This slight discrepancy may be due to the different D-Dimer assays used in the different studies, or sample sizes too small in those studies to capture D-Dimer false negative patients, a higher prevalence in our study of patients with chronic PE or already on anticoagulants, or variability and discordance among radiologists in their interpretations of CTPAs and diagnosis of pulmonary embolism [12, 36].

Of those patients who underwent CTPA, 34.6% did so without a D-Dimer done prior to CTPA examination. As discussed in the methodology section, it is not possible for us to comment on whether physicians formally risk-stratified these patients to be high-risk through application of the Wells score, and thus not candidates for rule-out through D-Dimer testing, or if the physicians simply chose to bypass an algorithmic clinical decision support aid and rely on their own assessment and clinical gestalt. What *can* be gleaned from these results is that the group of patients who had a D-Dimer prior to CTPA and the group of patients who did not actually had essentially similar positive yields for PE. Similarly, at the individual physician level, we did not observe a significant relationship between a physician’s degree of adherence with the practice of ordering a CTPA only after a positive D-Dimer and their individual CTPA ordering rate or their diagnostic yield.

While performing a CTPA on a patient who has a negative D-Dimer is demonstrably low yield, the benefit of mandating adherence to a Clinical Decision Support (CDS) or diagnostic algorithm predicated on the ordering of a D-Dimer prior to CTPA is a bit more ambiguous. The studies on the real-world impact of CDS and D-Dimer utilization on CTPA ordering patterns have yielded mixed results. Several landmark studies have shown a positive impact of increased D-Dimer utilization on the outcomes of interest of improved diagnostic yield and decreased CTPA utilization rate and volume [21, 37]. Other studies, however, have yielded less encouraging results with either no or negative impact of D-Dimer screening on diagnostic yield and ordering volume of CTPAs [38–40], demonstrating the limitations imposed by D-Dimer screening’s high false-positive rate and the application of such a screening test to a very low-risk population with a very low prevalence of PE. It should be noted that the initial landmark study introducing and validating the Wells score, and determining the prevalence of PE in the low-, intermediate- and high-risk group, was done on a population who had a 9.5–9.7% prevalence of PE [20, 41], indicating that the patient population in the study may have been a selected higher-risk population when compared to the unselected, undifferentiated ED patient presenting with any of the complaints of chest pain, dyspnea, syncope, generalized weakness and the myriad other subjective symptoms that may now spur a work-up for PE.

Studies have in fact shown that clinician gestalt may perform as well or better than a clinical decision rule or decision aid in predicting disease [42, 43]. As well, it is possible that the introduction of D-Dimer testing, done under the rationale – and supported by evidence – of allowing for a non-invasive pathway to screen low-risk patients for PE has, over time, had the unintended impact of creating a culture shift in emergency medicine in terms of lowering the cognitive load on physicians in the risk-stratification of patients for evaluation of PE. Therefore, it is possible that a far greater proportion of patients are now moved forward to screening with an objective diagnostic test, many of whom previously would have been excluded for PE – or never considered for PE in the first place – on purely clinical grounds. This may have compounded the well-known issues of applying a low-specificity screening test to a large population with a low disease prevalence.

## Limitations

There are several limitations to our study. Firstly and most importantly, it only examines the association between D-Dimer screening, and not the impact of adherence to risk-stratification through the Wells criteria, and the outcomes of diagnostic yield and utilization rate. While this was done by design, it does leave open the possibility that those CTPAs ordered without a prior D-Dimer were on patients whom the physician had formally risk-stratified as high-probability for PE and therefore not a candidate for rule-out through D-Dimer screening. However, if that were the case, the patients in whom no D-Dimer was ordered prior to CTPA would have a higher prevalence of PE, one that is closer to the 37.5% typically associated with high clinical probability patients [41]. Second, given that our study only looked at CTPA studies performed, we do not have data on how many CTPAs were avoided through negative D-Dimers. Third, we used a single D-Dimer cut-off value which was not adjusted for age. Fourth, within the group of ED physicians, there was a great deal of variability in terms of patient volume, patient acuity and volume of CTPA ordered by each physician. As well, we do not have data on the clinical characteristics of all ED patients seen by each individual physician, and are thus not able to correct for confounders arising from variability in the volume, acuity, and chief complaints of the patient populations seen by each MD. As such, any inferences made from direct comparisons between physicians and from unadjusted regression analyses must be interpreted with caution. Fifth we did not examine the issue of missed pulmonary embolisms. Missed diagnoses are often, due to their somewhat counterfactual nature, extremely challenging to detect within the confines of a retrospective observational study. However, large population-level studies have shown a rising incidence of PE without a concomitant fall in mortality [3], suggesting that all least some proportion of PEs diagnosed through more aggressive testing may be either false positives or of limited clinical significance [12, 44].

Finally, other limitations include those endemic to retrospective chart reviews, namely coding errors, biases introduced in the data abstraction process due to lack of blinding, and availability and quality of data.

## Conclusion

Overutilization of CTPA has often been attributed to inadequate adherence to established clinical decision rules, particularly those that integrate D-Dimer serology as an initial screening test for the evaluation of a patient suspected of PE. Physician education around established algorithms and institutionally mandated adherence to clinical decision supports, specifically those utilizing D-Dimer testing as an initial screening test for low-risk patients, have often been put forth as a simple solution to a complicated problem. The findings of this study, however, failed to show a positive impact of D-Dimer testing on either overall CTPA diagnostic yields or ordering rates. Mandatory adherence to diagnostic algorithms does have potential to improve ordering behaviour among physicians, particularly in terms of limiting the number of CTPAs ordered on patients with a negative D-Dimer. However, this strategy may prove – and has proven – to be fraught with its own challenges relating to the issue of D-Dimer testing’s high false-positive rate, as well as the overall challenges relating to monitoring, appraising, and providing feedback on physician ordering behaviour. A more individualized approach that combines audit-and-feedback and sensitization of individual physicians to their own level of risk tolerance and risk aversion may be needed in order to incur significant changes to the outcomes of interest of CTPA ordering volume and diagnostic yield.

## Data Availability

Public access to the dataset is closed. Administrative permission in the form of REB approval was required to access the raw data. The datasets included and/or analyzed during the current study are available from the corresponding author on reasonable request.

## Abbreviations

ED: Emergency Department;
CTPA: Computed Tomography Pulmonary Angiography
PE: Pulmonary Embolism
NACRS: National Ambulatory Care Reporting System
CTAS: Canadian Triage and Acuity Scale
CDS: Clinical Decision Support
IQR: Interquartile range
DVT: Deep vein thrombosis
